# Reconstructing Italy’s rural landscape before the Great Acceleration: A geospatial baseline from the *Catasto Agrario* (1929)

**DOI:** 10.12688/openreseurope.20343.1

**Published:** 2025-06-27

**Authors:** Filippo Brandolini

**Affiliations:** 1 Center for Sustainability Science and Strategy, Massachusetts Institute of Technology, Cambridge, Massachusetts, 02139, USA; 2 Università degli Studi di Milano Dipartimento di Scienze della Terra Ardito Desio, Milan, 20133, Italy

**Keywords:** Historic Land Use, Agroforestry, Regression Kriging, Demographic dynamics, Rural Heritage

## Abstract

This paper presents a geospatial dataset detailing the rural landscape of Italy in the 1920s, based on the Catasto Agrario 1929 survey. The dataset integrates data from the survey into a Geographic Information System (GIS), providing insights into land use and land cover (LULC), demographic characteristics, livestock distribution, crop yields, and precipitation patterns. Historical data have been digitised using Optical Character Recognition (OCR) and organised into a vector format, capturing the administrative boundaries of Italy's provinces as they were in the 1920s. By documenting Italy’s rural landscape just before the onset of the Great Acceleration (ca. mid-20th century CE) the dataset offers a critical historical baseline for analysing long-term socio-environmental transformations. The research aims to facilitate future studies on the environmental impacts of Italy’s rural transitions, offering an open-access resource that enables comparisons between past and present landscapes. It highlights the role of traditional agricultural practices, such as agroforestry, which were widespread before the shift towards modern monoculture systems. This dataset holds potential for applications in environmental sciences, historical geography, and heritage studies, providing a foundation for exploring sustainable agricultural practices and the enduring effects of rural depopulation and land-use change.

## Introduction

In recent decades, human activities have intensified considerably, resulting in notable changes to the landscape ^1^. These changes are associated with significant ecological consequences, including alterations in land use and vegetation, soil erosion, desertification, river dynamics, and more. Land Use and Land Cover (LULC) and their transformations are key variables that substantially affect environmental processes ^2^. LULC change datasets are crucial for environmental and resource management ^3^, allowing for precise monitoring of land use changes such as deforestation ^4^, urban expansion ^5^, desertification ^6^, and agricultural intensification ^7, 8^. These datasets are essential for sustainable resource management. In environmental modelling, LULC change data supports the prediction of future land use scenarios and the assessment of ecosystem impacts, thereby facilitating biodiversity conservation, water resource management, and mitigation of geomorphological hazard enhanced by climate change ^9, 10^.

Most LULC datasets rely on information gathered from satellite constellations like MODIS ^11^, Sentinel, and Landsat ^12^. Despite their high resolution and spatial coverage ^13^, a limitation remains their diachronic temporal resolution. The use of satellite data for studying LULC changes began in the early 1970s with the launch of the first Landsat satellite in 1972, marking the start of continuous and systematic Earth observation from space, providing valuable data for LULC studies ^14^. However, most significant LULC changes started at least a century earlier, around the mid-19th century, when urbanisation and industrialisation deeply impacted rural landscapes and ecosystems. To understand the impact of pre-1970s changes on climate and environments, it is crucial to integrate data from modern satellites with historical sources like aerial images, cadastral maps and historic cartography ^15– 18^. Landscape archaeology has the potential to fill this gap in the data by interpreting the spatial and chronological complexity of landscapes through two main tools: Historic Landscape Characterisation (HLC) and Historic Land-Use Assessment (HLA) ^19^. These methodologies introduce time-depth in landscape studies by assessing earlier patterns from historic or archaeological mappings, allowing an appreciation of past changes to guide future transformations ^20^. The integration of HLC and HLA within GIS (Geographic Information Systems) technologies ^21, 22^ underscores the importance of these landscape archaeological methodologies. They create invaluable datasets about past LULC and facilitate the interplay with other disciplines such as ecology ^23, 24^, geomorphology ^25, 26^, and geospatial statistics ^27, 28^.

The rural landscape of Europe underwent dramatic changes throughout the 20th century, largely driven by the socio-economic transformations associated with the Great Acceleration, a period of rapid industrialisation and agricultural intensification that began after the 1950s ^29, 30^. These transformations had profound environmental, cultural, and socio-economic impacts, emphasising the need for quantitative analysis to predict future scenarios in the face of ongoing global changes.

Over the last 7 decades, Italy's landscape has seen significant shifts, with urbanisation in the lowlands and forest expansion in the uplands standing out as the most notable trends ^31, 32^. These changes are closely linked to wider socio-economic factors such as rural depopulation, agricultural intensification, and industrialisation ^33^. Particularly after World War II (WWII), socio-economic dynamics triggered a profound transformation of Italy's rural landscape, which had previously been shaped by a long-standing historical rural tradition (Sereni, 1961). Understanding the LULC changes both before and after WWII is crucial for assessing the extent of this transformation in Italy's rural landscape.

Satellite data covers only the period from the 1970s to the present day, while historic aerial images can track LULC changes back to the 1950s. For earlier decades, information about landscape changes can be retrieved from historical cartography, cadastral maps, or land registries. However, these sources have often never been digitised, and even in cases where sporadic digital datasets exist, they often lack geospatial references, making their use in computational analysis challenging. Linking these invaluable sources of information with a spatial dimension facilitates the integration of historical data with LULC datasets developed using aerial images and satellite observations, allowing the reconstruction of human-environment interactions throughout the entire 20th century CE and even before.

The results presented here are based on the *Catasto Agrario 1929*, a survey conducted by the Italian National Institute of Statistics (Istituto Nazionale di Statistica - ISTAT) between 1928 and 1930 and it covers the whole Italian territory. This survey produced a volume for each province of Italy, offering detailed descriptive data on the Italian rural landscape in the 1920s. It serves as a comprehensive inventory, providing data on agricultural and forest areas, lands dedicated to individual crops, average crop yields per hectare plus other invaluable information such as the number of workers employed in agriculture, livestock, monthly precipitation rates, both at municipal and provincial levels ^34^.

The information retrieved from the Catasto Agrario was organised and georeferenced into a GIS vector dataset, providing an invaluable resource on Italy’s economic and environmental conditions in the 1920s. The dataset includes not only land use and land cover (LULC) data, but also information on demography, livestock, yield rates, and precipitation rates at the provincial level. It is intended to serve as a baseline for future research in both environmental and economic sciences, facilitating a deeper understanding of rural transformations in Italy over the past century.

## Methods

Data from the *Catasto Agrario* were digitised using a semi-automated workflow and subsequently integrated into a GIS framework to produce vector-based geospatial datasets. A standardised and carefully designed protocol was employed throughout the process to maintain consistency and ensure the reliability of the final dataset.

The initial phase focused on extracting data from the original rural registry. Digital copies of the *Catasto Agrario* volumes were obtained from the official ISTAT repository ^35^. Optical Character Recognition (OCR) was carried out using FineReader PDF 16 ^36^. This enabled the semi-automated extraction of tabular information, which was subsequently converted into spreadsheet format (.csv) ^37^. The second phase involved constructing an empty geospatial dataset representing the configuration of Italy’s provinces as they existed in 1929. Over the past 70 years, numerous changes have affected Italy’s administrative boundaries; whereas today the country is divided into 107 provinces, in 1929 there were only 91, with several subsequent subdivisions accounting for the increase. It was therefore essential to identify and adjust areas that no longer corresponded to the historical territorial divisions. Each volume of the *Catasto Agrario* included a map depicting the administrative boundaries at the time of data collection. These maps were georeferenced in QGIS ^38^ following a 'backdating approach' ^39, 40^ to detect discrepancies between historical and contemporary boundaries and place names. In parallel, modern provincial vector layers were sourced from the National Geodatabase ^41^ and edited to reconstruct the 1929 territorial framework. The resulting layers were exported in the Geopackage (GPKG) format ^42^, a standardised, platform-independent format selected to facilitate widespread reuse of the dataset. The third phase entailed transferring the information extracted via OCR from the 1920s rural registry into the empty GPKG files. To facilitate this process, a Python script ^43^ was developed to automatically retrieve data from the OCR-generated spreadsheets, create the necessary table fields, and systematically organise the information for each province. This script can be run through the QGIS Python interface to ensure consistency in database development. It is based on two widely used Python libraries for data manipulation and geospatial data management: Pandas and GeoPandas. Pandas is a powerful Python library primarily used for data manipulation and analysis. It provides data structures like DataFrames and Series, which allow for efficient handling of structured data, such as tabular data from spreadsheets or databases. Pandas is particularly useful for data cleaning, filtering, aggregation, and transformation tasks, making it a go-to tool for data scientists and analysts working with large datasets ^44^. GeoPandas extends the capabilities of Pandas to handle geospatial data. It allows for the manipulation of geometric objects, such as points, lines, and polygons, and integrates spatial operations into the familiar Pandas framework. With GeoPandas, spatial joins, projections, and geospatial analyses can be performed directly on geospatial data, making it a valuable tool for tasks involving mapping, GIS, and spatial data science ^45^. The resulting output is an updated GPKG file detailing the rural setting of Italy in the 1920s. As data is added to or removed from the spreadsheets, the Python script automatically updates the GPKG file. The final step involves dataset validation. To minimise topological errors, the QGIS Topology Checker plugin was used to identify potential spatial inconsistencies, including overlaps, slivers, and duplicates. These issues were subsequently corrected using the v.clean module from the GRASS software suite ^46^.

The EU CORINE Land Cover (CLC) nomenclature ^47^ was adopted to classify the LULC types recorded in the *Catasto Agrario*. The CLC system offers a hierarchical framework of classes, accompanied by detailed descriptions and methodologies for visual interpretation, thereby promoting consistency across mapping activities. The LULC categories applied in this dataset include agroforestry (CORINE class 2.1.1), fruit tree plantations (2.2.2), pasture (2.3.1), arable land (2.4.4), grassland (3.2.1), managed forest (3.1.1), and mixed forest (3.1.3). In addition, two supplementary classes were incorporated: uncultivated productive area and unproductive area. The former corresponds to areas identified in the *Catasto Agrario* as *Incolti produttivi*, referring to land that was potentially cultivable but not under active use at the time of survey. The latter encompasses all remaining provincial areas not explicitly classified, such as geologically unproductive zones (e.g., rock outcrops), water bodies (e.g., lakes, rivers), and infrastructure (e.g., buildings, roads, railways). The adoption of these categories was intended to closely mirror the original structure of the *Catasto Agrario*. For example, woodland areas were typically recorded without specifying dominant tree species (i.e., broadleaf or coniferous), which justified their classification as mixed forest. Conversely, chestnut groves, when explicitly identified, were categorised separately as managed forest.

Mean precipitation rates for 1929, recorded in the *Catasto Agrario* for most Italian provinces, were organised in tabular form, with monthly precipitation values documented at weather stations within provincial territories ^37^. This valuable rainfall data from the 1920s was digitised into a GIS using a systematic approach. For each province, precipitation rates from individual stations were extracted using the same OCR method mentioned earlier and compiled into a dedicated spreadsheet. Since the exact locations of the weather stations are unknown, and the *Catasto Agrario* provides no coordinates, the centroid of each place name was used to create a vector point layer of meteorological stations in GPKG format. This ‘empty’ point layer was then populated with data from the spreadsheet using a Python script like the one used for the LULC GPKG dataset previously described.

Geostatistical analysis of the 1920s precipitation data was conducted using regression kriging ^43^, a widely employed method for rainfall prediction ^48– 50^. Key factors such as distance from the coastline, elevation, slope, and aspect were used in the analysis because they directly influence local climatic conditions and precipitation patterns. These variables help enhance the spatial accuracy of precipitation estimates by accounting for crucial geographic and topographic factors. As these factors have remained largely unchanged over the past 70 years, they provide a reliable basis for interpolating the 1920s precipitation rates recorded in the *Catasto Agrario*.

Distance from the coastline plays a crucial role in precipitation patterns, as proximity to large bodies of water increases local humidity and rainfall, particularly when moist air masses move inland. As the distance from the coast increases, moisture availability decreases, altering precipitation patterns ^50^. A raster file of the Euclidean distance from the Italian coastline was developed using the GRASS module r.grow.dist.

Elevation significantly influences precipitation due to orographic effects ^48^. As moist air rises over elevated terrain, it cools and condenses, leading to higher precipitation on windward slopes. In contrast, leeward slopes in rain shadows receive less precipitation. Including elevation in the model captures these terrain-driven precipitation patterns, particularly in areas with significant elevation changes ^51^. The DEM provided by the Italian Istituto Nazionale di Geofisica e Vulcanologia ^52^ was downscaled to 200 m and used as the elevation covariate in the regression kriging.

Slope plays a crucial role in how air masses interact with the landscape. Steeper slopes intensify orographic uplift, resulting in increased precipitation, particularly in regions where winds push air upward. In contrast, gentler slopes have a more limited effect on air movement and precipitation. Incorporating slope into the model helps better capture the relationship between terrain and atmospheric conditions ^49^.

Aspect, the direction a slope faces, also significantly influences local climate. Slopes that face prevailing winds or the sun can experience different microclimates compared to those facing away. For example, south-facing slopes receive more solar radiation, which impacts temperature and air movement, ultimately affecting precipitation patterns. Windward slopes, exposed to prevailing winds, tend to receive more precipitation, while leeward slopes, sheltered from the wind, receive less. Including aspect in the model allows for a more accurate representation of these directional effects on precipitation distribution ^50^.

Slope and aspect covariates were computed in Python, with slope in degrees and aspect on a 0–360° scale, based on DEM gradients. Terrain attributes—distance from the coastline, elevation, slope, and aspect—underwent imputation for missing data and standardisation to ensure consistency across features. The core analysis employed regression kriging via the PyKrige library ^53^. A multiple linear regression model was trained with terrain features as independent variables and precipitation values as the dependent variable. A grid matching the DEM resolution was generated for spatial prediction, and the regression kriging model was applied to estimate precipitation values across the region of interest. These predictions were output as a GeoTIFF file, providing a spatial representation of the 1920s precipitation distribution.

## Results

**Figure 1.**
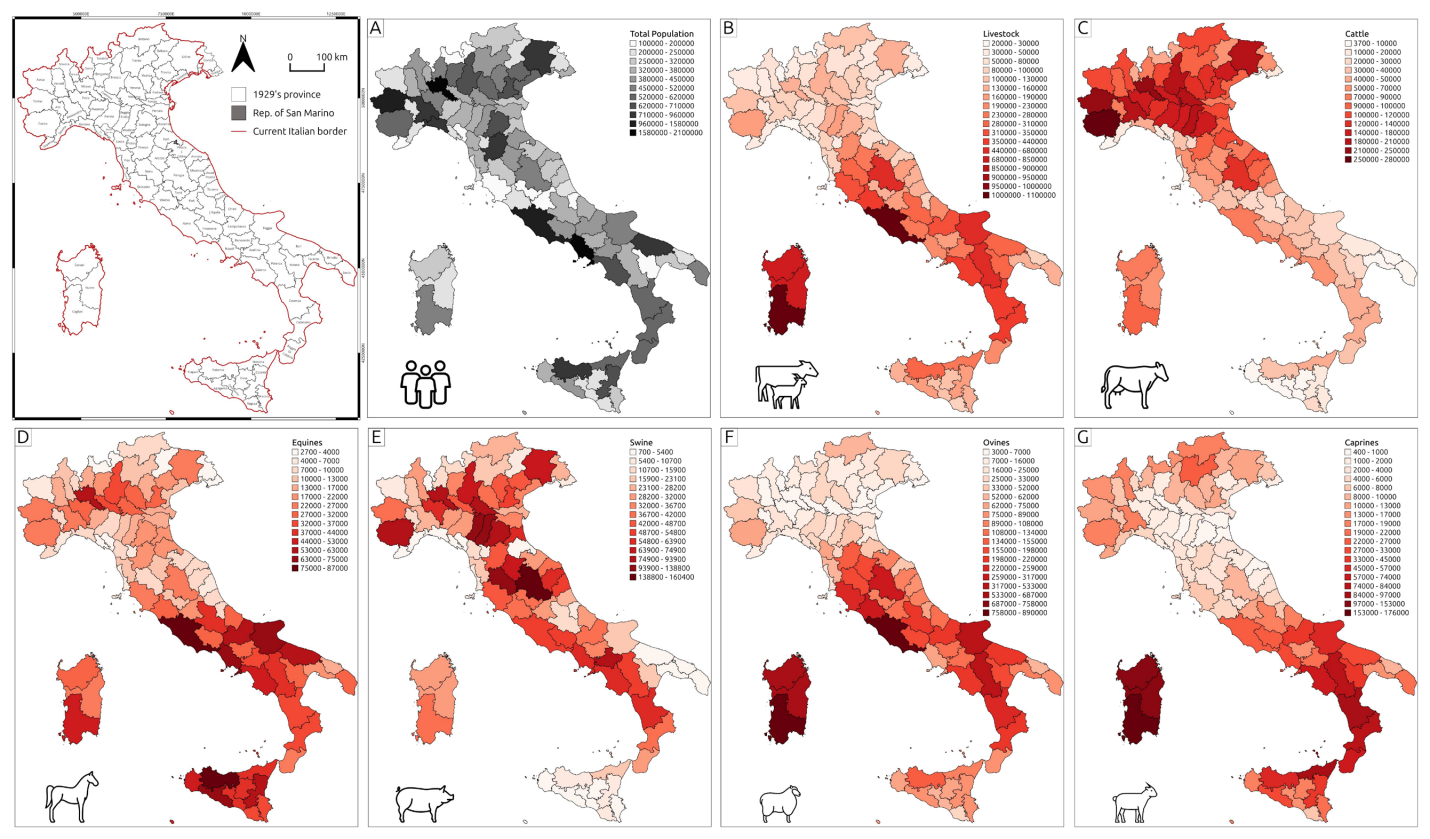
Thematic maps representing demographic and livestock data collected in the Catasto Agrario (1929). **A **– Total population; **B** – Livestock; **C** – Cattle; **D** – Equines; **E** – Swine; **F** – Ovines; **G** – Caprines.

**Figure 2.**
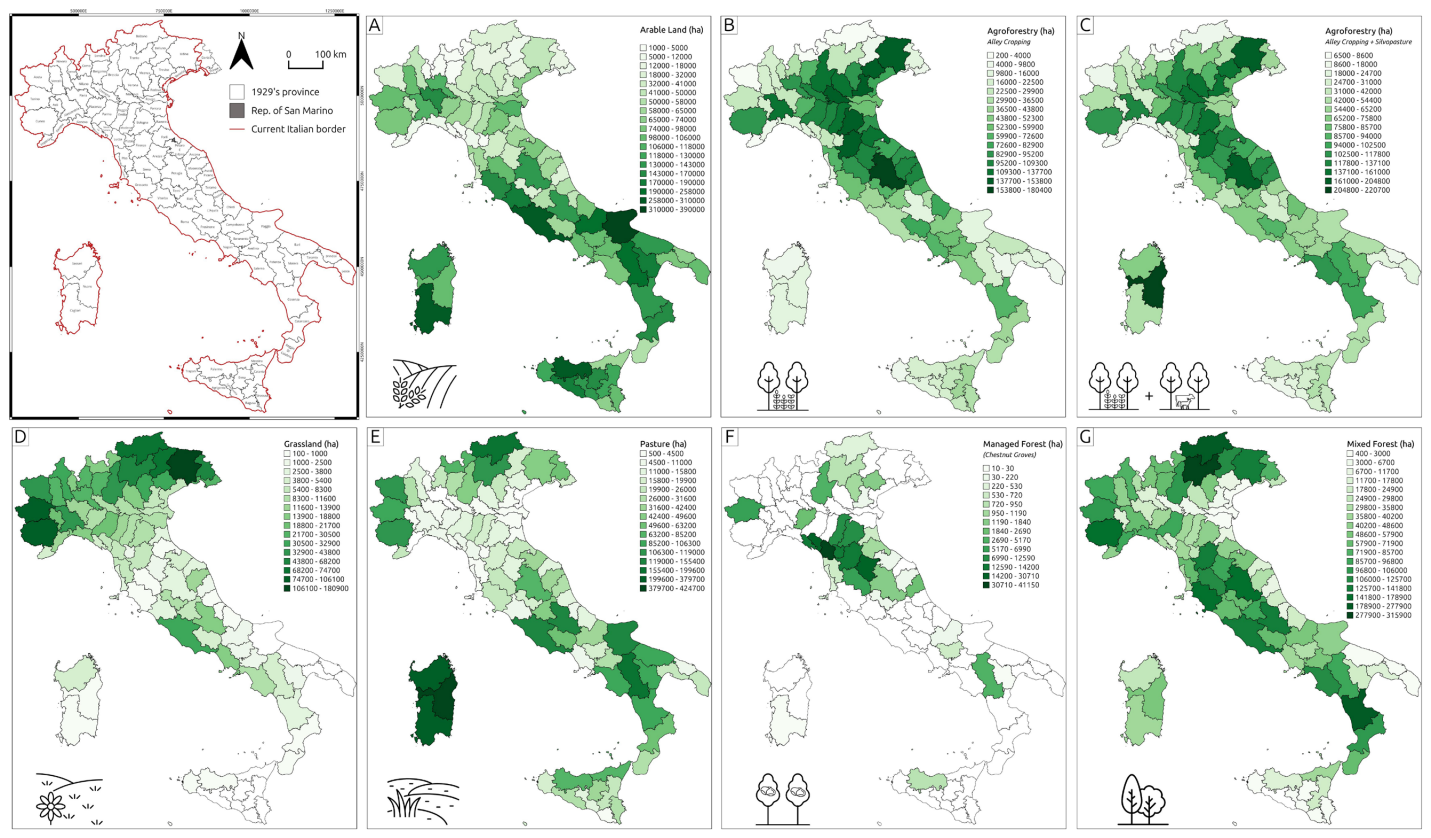
Thematic maps representing land use and land cover (LULC) types derived from the Catasto Agrario (1929). **A** – Arable land; **B** – Agroforestry (Alley cropping); **C** – Agroforestry (Alley cropping and Silvopasture); **D** – Grassland; **E** – Pasture; **F** – Managed forest; **G** – Mixed forest.

**Figure 3.**
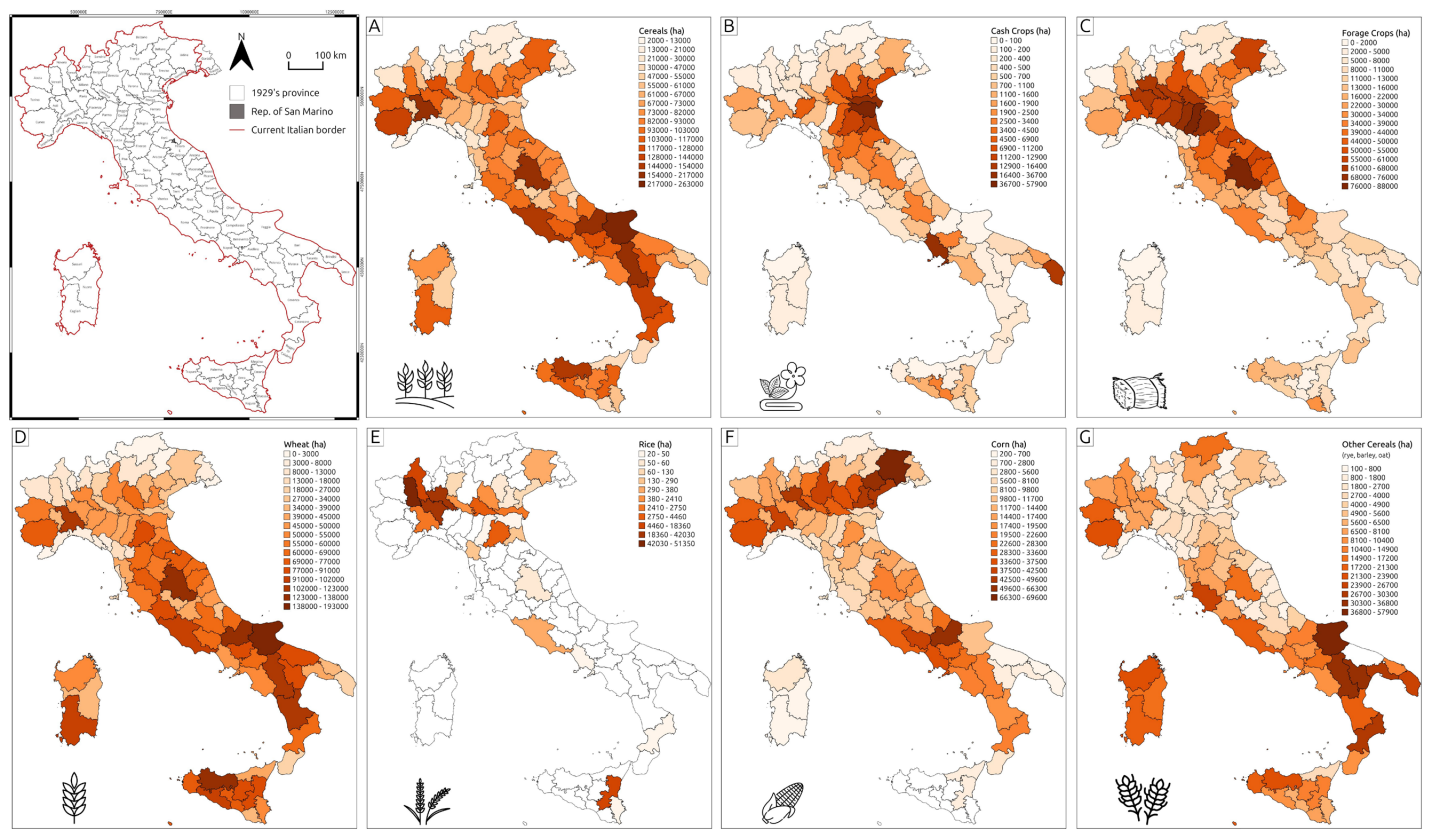
Thematic maps representing crop type distributions recorded in the Catasto Agrario (1929). **A** – Cereals; **B** – Cash crops; **C** – Forage crops; **D** – Wheat; **E** – Rice; **F** – Maize; **G** – Other cereals.

The geospatial dataset resulting from the above mentioned operations consists of a multipolygon vector layer representing the 91 provinces of Italy in the 1920s. The vector layer is linked to an attribute table containing 106 entries, reflecting the key characteristics of the Italian historic rural landscape prior to WWII (Table 1 in Extended data). The units of measurement used are hectares (ha) for spatial extent and quintals (q) for weight-based quantities. The attributes were recorded in the dataset according to the original structure of the *Catasto Agrario*, including demographic information ( Figure 1), livestock ( Figure 1), LULC ( Figure 2), detailed crop types, their extent ( Figure 3), and yield rates (q/ha) (Table 1 in Extended data).

**Figure 4.**
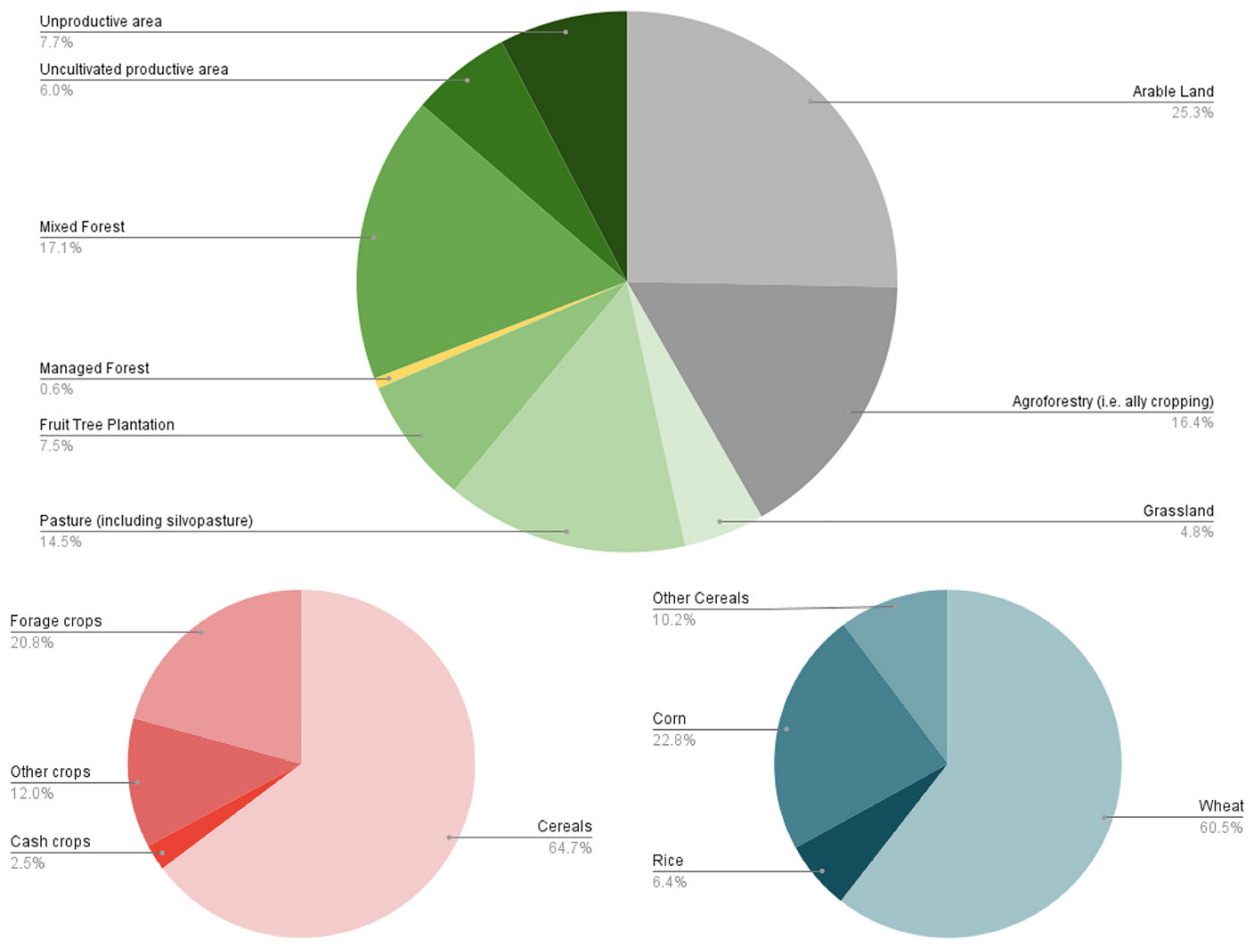
Overview of Italy’s historic rural landscape in the 1920s. **A** – Distribution of LULC types; **B** – Percentage of farmland allocated to each crop type; **C** – Percentage distribution of specific cereal types ('Other cereals' includes rye, barley, and oats).

The geospatial dataset has been analysed using some basic exploratory data analysis tools to summarise its key characteristics. Figure 4 illustrates that, in the 1920s, Italy's rural landscape was predominantly shaped by agropastoral activities, with monoculture arable land being the most widespread agricultural system ( Figure 2, A). Agroforestry – the land management system integrating trees and shrubs into agricultural landscapes alongside crops and/or livestock ^54^ – also played a significant role in the national agricultural economy. The two most common agroforestry practices were *alley cropping*, which involves planting rows of trees in agricultural fields with alleys between the rows for growing crops, and *silvopasture*, where trees, forage, and livestock grazing coexist on the same patch of land. To highlight the relevance of agroforestry in the 1920s national economic systems, Figure 2 displays both situations: the total extent of alley cropping in each province ( Figure 2, B) and the total area allocated for agroforestry practices ( *alley cropping + silvopasture* - Figure 2, C). Conversely, Figure 1 reflects the structure of the *Catasto Agrario*, where silvopasture was classified as a subtype of the pasture class.

When excluding arable land (25.3%) and agroforestry (16.4%), several observations can be made about the remaining categories in Figure 1. Mixed forest, accounting for 17.1% of the total land use, constitutes a significant portion of the landscape. This suggests that forested areas, particularly mixed forests without specific details on tree species, play an important role in the region’s land cover. Pasture, including silvopasture (14.5%), also represents a notable land use, indicating the coexistence of livestock farming alongside natural or managed vegetation systems. Unproductive areas (7.7%) and uncultivated productive areas (6.0%) together make up 13.7% of the total land cover. The relatively high percentage of uncultivated but potentially productive land suggests the possibility of alternative land management practices. Unproductive areas, which include rock outcrops or infrastructure, signify parts of the landscape that do not directly contribute to agricultural or forestry activities. Fruit tree plantations (7.5%) reflect the importance of orchards and fruit production in the regions, contributing to agricultural diversity. Grasslands, covering 4.8% of the landscape, represent a smaller portion compared to other categories, likely serving as natural or semi-natural areas for grazing or biodiversity conservation. Lastly, managed forests, comprising only 0.6% of the total, indicate that actively managed woodlands, such as chestnut groves, cover a relatively small extent of the landscape.

Cereal cultivation, especially wheat, was the most common practice. In addition to wheat, the *Catasto Agrario* reported the spatial extent and annual production of rice and corn, while minor cereal production (such as rye, barley, or oats) was generally labelled as ' *Other Cereals*’. Additionally, at the national level, average yield rates indicate a slight difference in cereal production between monoculture and agroforestry systems. In many cases, the two systems were integrated within the same landscape, with agroforestry complementing monoculture practices ^55^. As displayed in Table 2, there were often no significant differences in production yields, and both systems contributed to maintaining overall agricultural productivity.

**Table 2.** National mean yield rates for each cereal type (wheat, rice, corn, others) in both monoculture (M) and agroforestry (AF) systems.

Wheat Yield (q/ha)		Rice Yield (q/ha)		Corn Yield (q/ha)		Other Cereals Yield (q/ha)	
M	AF	M	AF	M	AF	M	AF
14.48	14.50	41.15	40.29	16.05	16.24	12.48	12.49

As displayed in Figure 2, the primary tree species utilised in agroforestry systems were fruit trees, with regional variations such as apple, pear, apricot, peach, walnut, and cherry. Mulberry was also commonly used, particularly in agroforestry vineyard systems known as *coltura promiscua*, which refers to the traditional combination of trees, vines, and arable crops ^55^. The use of citrus trees in polyculture was limited, whereas olive trees were extensively employed in southern Italy in agro-sylvo-pastoral systems, where they were integrated with pastureland, shrubs, and agricultural fields.

The 1929 precipitation rates were collected in a point vector layer associated with an attribute table that reports the number of rainy days and the rainfall (measured in mm) for each month in 1929 at each of the 398 weather stations ( Table 3). The result of the regression kriging applied to the weather station dataset is a output representing the precipitation rates in Italy during the 1920s ( Figure 6). To evaluate the reliability of the kriging results, several validation methods were employed. Cross-Validation Mean Absolute Error (MAE), Mean Squared Error (MSE), and R² score were used to assess the performance of the kriging model in predicting precipitation rates ( Table 4). MAE measures the average absolute difference between predicted and actual values, providing a straightforward indicator of model accuracy. MSE, which squares the errors, penalises larger deviations more heavily, offering insight into the presence of significant outliers. The R² score, or coefficient of determination, measures the proportion of variance in the data that the model can explain, giving an overall indication of model fit ^56^. The MAE, with a value of 366.64, represents the average absolute difference between predicted and actual precipitation values, offering a clear measure of accuracy. The MSE, calculated as 205,246.11, squares the errors to penalise larger deviations, highlighting the influence of outliers. The R² score is 0.21, indicating that the model explains 21% of the variance in the precipitation data. These methods were selected to validate the kriging model’s ability to predict spatially variable precipitation with precision.

**Table 3.** Example of the attribute table associated with the point vector layer of precipitation data for 1929. The weather stations of the Umbria Region (UMB) are shown. For each month, the total precipitation (mm) and the number of rainy days (d) are reported, along with the overall amount of rainfall and the total number of rainy days.

Name	Reg	Jan (mm)	Jan (d)	Feb (mm)	Feb (d)	Mar (mm)	Mar (d)	Apr (mm)	Apr (d)	May (mm)	May (d)	Jun (mm)	Jun (d)
TORGIANO	UMB	45	5.3	62	7.3	74	9.4	81	9.2	53	7.8	60	5.7
VALFABBRICA	UMB	55	6.6	64	7.4	62	7.5	90	7.6	76	8.8	56	4.7
CASTIGLIONE DEL LAGO	UMB	57	6.9	55	6.4	73	7	70	7.3	52	7.2	47	4.3
MASSA MARTANA	UMB	79	5.2	72	5	93	6.3	110	8	98	7	66	4
GUALDO TADINO	UMB	96	8.8	121	9.6	110	12	157	12.2	114	11.4	100	7.7
Name	Reg	Jul (mm)	Jul (d)	Aug (mm)	Aug (d)	Sep (mm)	Sep (d)	Oct (mm)	Oct (d)	Nov (mm)	Nov (d)	Dec (mm)	Dec (d)
TORGIANO	UMB	24	2.4	26	3.1	68	5.6	71	6.7	76	9.9	46	6.8
VALFABBRICA	UMB	16	2.4	23	2.2	41	5.7	59	6.6	85	9.2	71	7.6
CASTIGLIONE DEL LAGO	UMB	26	2.1	31	2.6	61	3.6	87	5.9	86	8.3	71	7.3
MASSA MARTANA	UMB	33	2	53	2.8	76	4.6	119	5.4	131	7.4	78	6.8
GUALDO TADINO	UMB	37	3.7	65	5	132	8.3	114	9	163	11.7	176	11.2

## Discussion and conclusions

The dataset and maps presented here provide a visual overview of Italy's rural landscape during the 1920s. The demographic data ( Figure 1, A) illustrates the population distribution across the Italian regions. Similarly, the livestock data offers a detailed view of the spatial distribution of various livestock types throughout Italy during the 1920s ( Figure 1, B–G).

**Figure 5.**
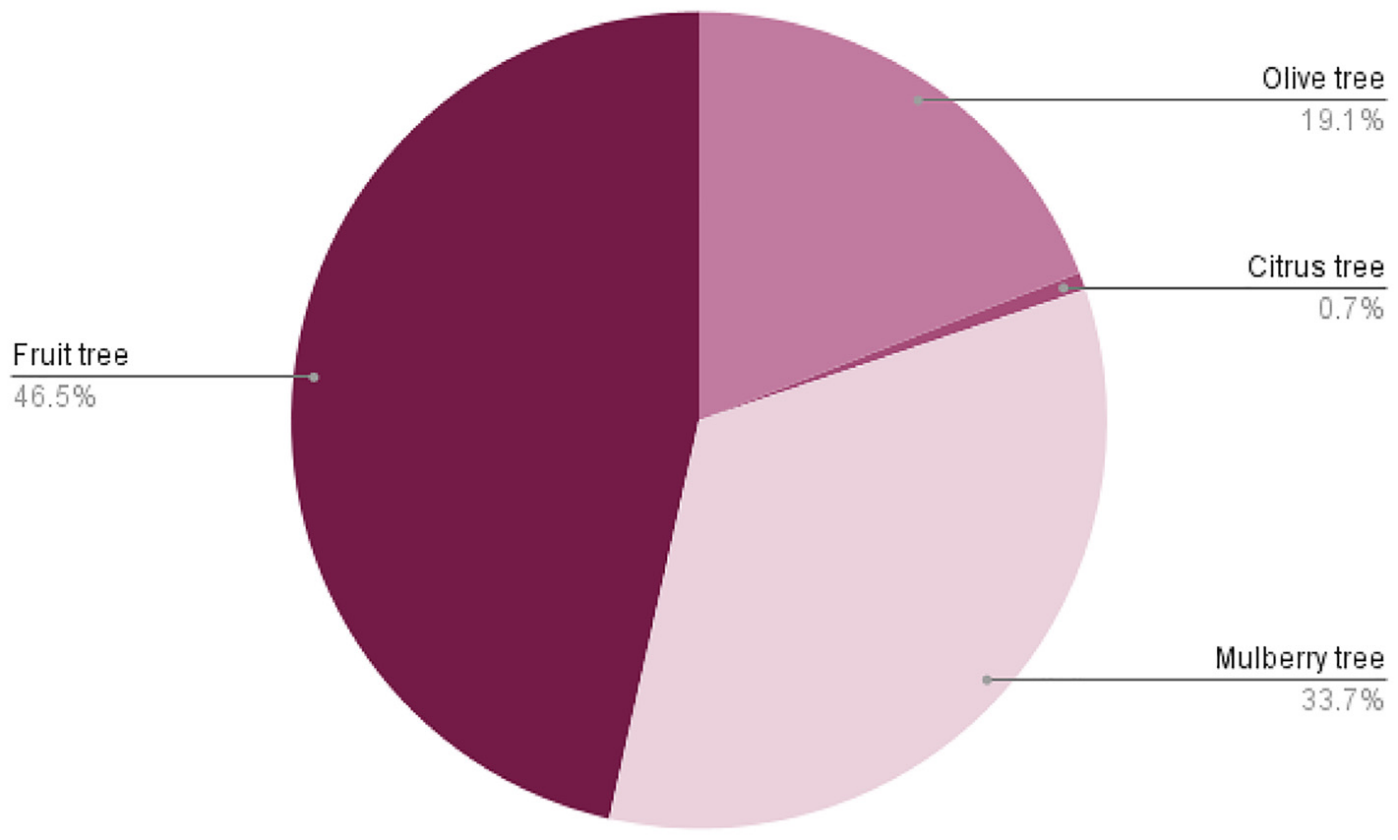
Tree species employed in agroforestry systems, highlighting regional variation in species used for alley cropping, silvopasture, and mixed cropping systems.

In the context of LULC setting ( Figure 2), the prominent role of agroforestry during this period is particularly significant, especially considering its gradual abandonment in favour of modern industrial monoculture agriculture from the 1950s onwards ^57^. In provinces of northern Italy, agroforestry was integral to the rural economy of the 1920s ( Figure 4). The most common polyculture system, known as ' *coltura promiscua*', involved the integration of crops with permanent mulberry trees, which also supported vines for grape cultivation. The exceptionally high occurrence of mulberry trees ( Figure 5) was closely linked to the silk industry (prominent since the 1920s), as their leaves were used to feed silkworms. The decline of agroforestry after WWII was driven by the advent of mechanisation and the rise of industrial monoculture in national agriculture, influenced by evolving socio-economic dynamics ^58^. This radical shift in the national rural LULC setting had potential environmental consequences, as agroforestry is regarded as one of the most sustainable agricultural strategies for supporting biodiversity ^59^, enhancing carbon sequestration ^60^, mitigating soil erosion ^26^, improving the water balance, reducing wildfire risks, and preserving traditional agricultural landscapes and rural knowledge ^61^. The widespread abandonment of agroforestry in Italy also had detrimental effects on the cultural values of the landscape. In addition to environmental degradation, the progressive loss of farmers’ expertise in these rural practices led to substantial physical changes in the regional landscape heritage ^62^.

**Figure 6.**
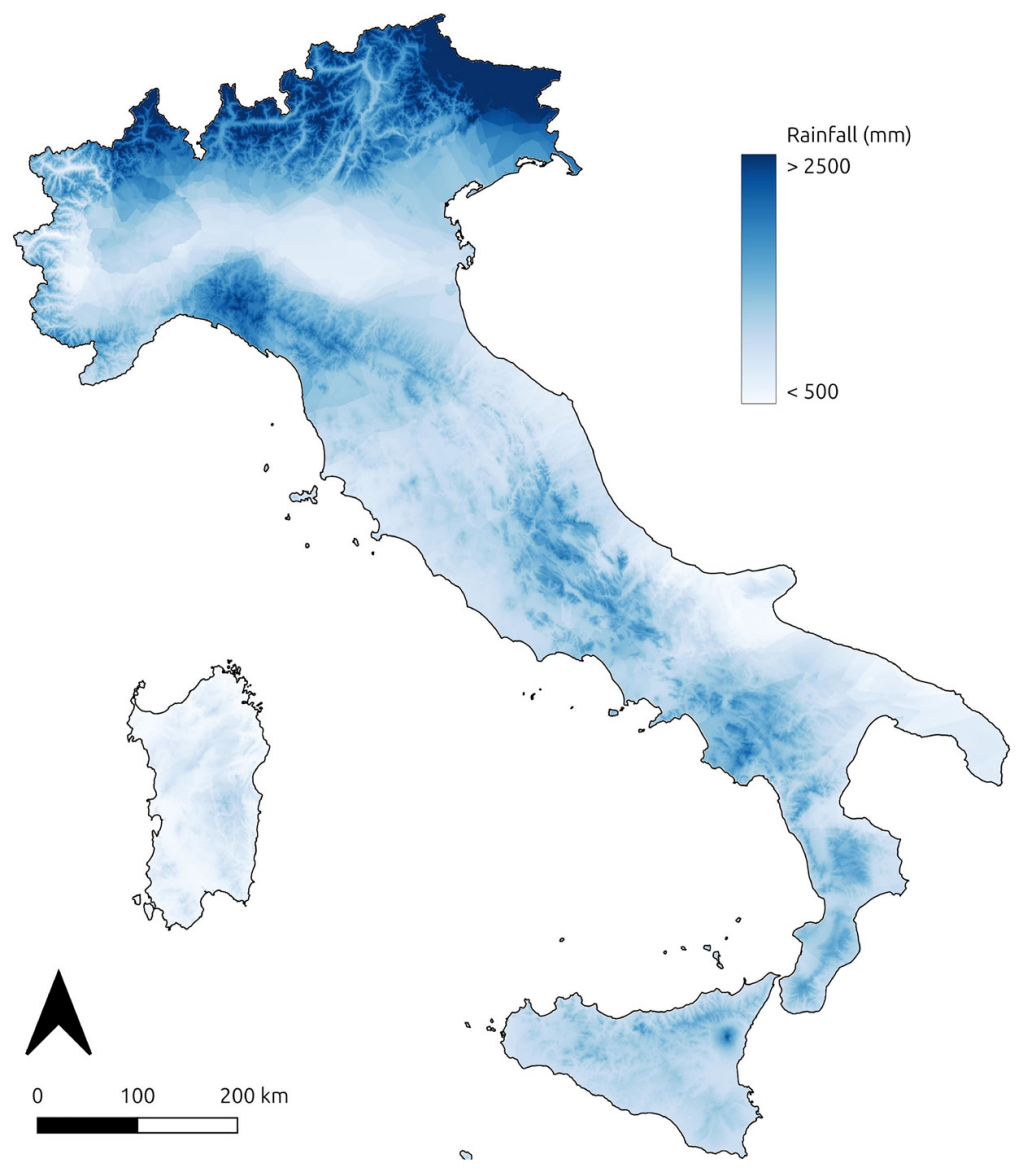
Regression kriging results showing spatial distribution of 1920s precipitation rates across Italy, based on weather station data digitised from the Catasto Agrario (1929).

Regarding Figure 6, the validation method for the geostatistical analysis of the 1920s precipitation data highlights uncertainties within this model ( Table 4). The R² score, along with the relatively high MAE and MSE, suggests that the model is not performing well and has limited predictive power. Since the primary goal of this research was to develop a dataset associated with an output to help visualising precipitation distribution in Italy during the 1920s, the kriging performance was not explored further. For future reuse of the precipitation rate data in climatic or environmental modelling, it will be essential to improve the analysis by incorporating more predictive variables and exploring alternative approaches.

**Table 4.** Performance metrics for kriging model validation, including Mean Absolute Error (MAE), Mean Squared Error (MSE), and R² score.

Kriging performance validation method	Value
Mean Absolute Error	363.41
Mean Squared Error	203320.59
R² score	0.2236

Finally, the dataset provided here holds significant potential for reuse in future studies, offering valuable insights into a range of research areas. For studies on LULC, these historical data can serve as a baseline for understanding how rural landscapes have evolved over the past century, especially in response to agricultural industrialization and urbanisation. The demographic pressure that began in the 1950s played a pivotal role in driving both urban expansion and the intensification of agricultural practices. In response to increased food demand, agricultural systems transitioned from extensive models, such as agroforestry, to more intensive methods of cultivation. This shift was further catalysed by the agrarian reforms of the 1940s and 1950s, which, beyond addressing socio-economic demands, were also politically motivated to increase productivity. These reforms transformed Italy's agricultural landscape, particularly in rural areas, by promoting intensification and modernising agricultural structures ^63^. By comparing past and present land use, researchers can identify trends in landscape transformation and their environmental, economic, and social impacts. In the context of sustainable agricultural practices, the data can inform research on the effectiveness of pre-industrial traditional systems which are recognized for their benefits in supporting biodiversity, enhancing carbon sequestration, and maintaining soil health ^64^. Understanding the factors that led to the abandonment of such systems may also help guide contemporary efforts to integrate sustainability into modern agricultural strategies.

From a heritage preservation perspective, the data can play a crucial role in understanding the cultural significance of traditional rural practices and their impact on regional identities. By documenting landscape changes and the loss of pre-industrial systems, this dataset may support efforts to preserve cultural landscapes, rural knowledge, and agricultural heritage that have been gradually eroded over time ^28^.

Additionally, demographic data can help researchers track population changes over the last century. Since the 1950s, the depopulation of certain regions and the internal migration of people from remote areas to more industrialized and urbanized zones have been notable phenomena on the Italian peninsula ^65^. The geospatial dataset presented here serves as a valuable resource for modeling demographic dynamics in Italy throughout the 20th century. Similarly, the spatial distribution of livestock types can provide deeper insights into the shift from more sustainable, traditional livestock farming methods to modern intensive systems, which developed in response to socio-economic demands following WWII. Cattle farming, particularly for dairy and beef production, is a major contributor to greenhouse gas emissions in Italy ^66^. A diachronic analysis comparing the quantitative spatial distribution of livestock in the 1920s with contemporary data could aid in developing new sustainable practices.

In terms of policy development, the insights gained from this dataset can be instrumental in shaping initiatives aimed at revitalising traditional rural practices. Such policies could not only help to restore degraded landscapes but also foster rural development, support biodiversity, and enhance climate resilience. By drawing on lessons from the past, policymakers could create frameworks that balance economic viability with ecological sustainability, promoting a more harmonious relationship between agriculture and the environment ^67^. Overall, the dataset provides a rich resource for understanding historical land use dynamics and has wide-ranging applications for addressing contemporary challenges related to sustainability, rural development, and environmental conservation.

## Software

The OCR software ABBYY FineReader PDF 16 (A copyright license for the use of this proprietary software was obtained by the author). was used to extract information from scanned PDF files of the ISTAT *Catasto Agrario 1929*. The thematic maps ( Figure 1– Figure 3 and Figure 6) and the related geodatabase were edited using QGIS 3.42 Munster (free and open-source software). Script codes for building the dataset and performing kriging interpolation were developed using the Python 3.12.3 programming language.

## Ethics and consent

Ethical approval and consent were not required.

## Data Availability

Zenodo: Rhecast_ORE_2025_software data, https://doi.org/10.5281/zenodo.15526385 ^37^ This project contains the following underlying data: ORE_2025_Dataset.zip Data is openly available under the Creative Commons Attribution 4.0 International License (CC BY 4.0) Zenodo: Rhecast_ORE_2025_software data, https://doi.org/10.5281/zenodo.15526385 ^37^ This project contains the following underlying data: Extended_data.docx Data is openly available under the Creative Commons Attribution 4.0 International License (CC BY 4.0)

## Software availability

The source code developed for this research is openly available under the terms of the MIT License, an OSI-approved open-source license designed specifically for software. This license permits reuse, modification, and distribution with minimal restriction, provided that the original copyright and license terms are preserved.

- Source code available from: https://github.com/Filbra/ORE_2025
- Archived source code: https://doi.org/10.5281/zenodo.15389080
- License: MIT License
