# Supplementary material for: Reconstructing Italy’s rural landscape before the Great Acceleration: A geospatial baseline from the Catasto Agrario (1929)
Source: Open Res Eur. 2025 Jun 27;5:169. [Version 1] doi: 10.12688/openreseurope.20343.1 (PMC12361881; doi:10.12688/openreseurope.20343.1)
Supplement: Peer review history

# Reviewer report: Reviewer response for version 1

**DOI:** 10.21956/openreseurope.22012.r56259

**Published:** 2025-08-20

**Authors:** Christos Chalkias

**Affiliations:** 1 Harokopio University, Kallithea, Greece

The paper “Reconstructing Italy’s rural landscape before the Great Acceleration” presents a novel geospatial dataset derived from the Catasto Agrario survey (1929) that digitises historical records and integrates them into a GIS framework. It reconstructs land use and land cover (LULC), demography, livestock distribution, crop yields and rainfall patterns in the Italian provinces in the 1920s. This work provides a rare pre-satellite baseline for analysing socio-environmental changes before the mid-20th century and represents an open-access resource for interdisciplinary research in environmental sciences, historical geography and cultural heritage studies. The strengths of the study lie in its innovative contribution, which fills a critical temporal gap in the LULC datasets, and in its methodological rigour. By combining OCR, GIS processing and regression kriging, the authors manage to process complex historical data and generate thematically rich geospatial data. The open accessibility of the dataset further enhances its relevance and potential impact and encourages reuse in future studies on landscape change, sustainable agriculture and rural heritage preservation. However, there are some limitations. The regression kriging used to reconstruct the precipitation data has low predictive power (R² = 0.21), which is acknowledged by the authors but not addressed in detail. Although the semi-automatic OCR process is efficient, it carries a risk of transcription errors despite the validation steps. In addition, the report could provide a deeper examination of the socio-economic drivers of landscape change beyond general references to industrialisation and rural depopulation. Given the complexity of the transition from raw historical documents to GIS-based thematic maps, a schematic diagram illustrating this data flow — from the Catasto Agrario inputs through digitisation, georeferencing and data processing to the final results — would greatly facilitate the reader's understanding and support reproducibility. It is also recommended that the authors explicitly summarise the limitations and assumptions of their approach in the discussion section. This would help future users of the dataset to better understand possible sources of uncertainty and the scope of applicability of the results. Overall, this is a well-executed and highly valuable contribution that forms the basis for further diachronic studies of Italy’s landscapes. With minor methodological clarifications, the addition of a workflow diagram and a concise summary of the assumptions and limitations in the discussion, it could have a strong impact in both academic and applied contexts.

Is the study design appropriate and does the work have academic merit?

Yes

Is the work clearly and accurately presented and does it cite the current literature?

Yes

If applicable, is the statistical analysis and its interpretation appropriate?

Partly

Are all the source data underlying the results available to ensure full reproducibility?

Yes

Are the conclusions drawn adequately supported by the results?

Yes

Are sufficient details of methods and analysis provided to allow replication by others?

Partly

Reviewer Expertise:

Applied Geography, Geoinformatics, GIS, Historical GIS

I confirm that I have read this submission and believe that I have an appropriate level of expertise to confirm that it is of an acceptable scientific standard, however I have significant reservations, as outlined above.

# Reviewer report: Reviewer response for version 1

**DOI:** 10.21956/openreseurope.22012.r56668

**Published:** 2025-08-18

**Authors:** Ebrahim Jahanshiri

**Affiliations:** 1 Crops For the Future - National Institute of Agricultural Botany, Cambridge, UK

The article presents a geospatial dataset reconstructing Italy's rural landscape in the 1920s, derived from the *Catasto Agrario* 1929 survey conducted by ISTAT. The dataset integrates digitized historical data on land use and land cover (LULC), demographics, livestock, crop yields, and precipitation into a GIS framework, using vector formats aligned with 1929 provincial boundaries. Employing OCR for data extraction, Python scripting for automation, and regression kriging for precipitation interpolation, the work creates an open-access baseline for analyzing socio-environmental changes before the "Great Acceleration" post-1950s. It emphasizes traditional practices like agroforestry, provides thematic maps and exploratory analyses, and discusses applications in environmental sciences, historical geography, and sustainability studies. Data and code are available on Zenodo and GitHub under open licenses, with limitations in kriging performance openly acknowledged.The work contains a logical structure from introduction to methods, results, and discussion, supported by 67 citations that effectively blend current literature on LULC, GIS, and landscape archaeology (e.g., references to CORINE data and kriging methods). The study design is appropriate and academically sound, as it addresses a critical gap in pre-1970s LULC data by creating a reproducible, open-access dataset that facilitates interdisciplinary comparisons between historical and modern landscaps, particularly in the context of Italy's rural transformations and sustainable agriculture. Methods are detailed sufficiently for replication, including OCR tools, QGIS workflows, python libraries (Pandas, GeoPandas, PyKrige), and validation steps like topology checking, with all scripts and data openly available, ensuring full reproducibility.

 The statistical analysis via regression kriging is appropriately applied and interpreted, incorporating relevant covariates (e.g., elevation, slope) and validation metrics (MAE, MSE, R²), while transparently noting limitations like the low R² (0.22), which suggests room for refinement but does not undermine the dataset's utility as a visualization tool. Conclusions are supported by results, highlighting agroforestry's historical dominnce and the dataset's potential for reuse in policy and heritage studies, without overstatement. Overall, I have no major criticisms requiring revision; it exemplifies best practices in open data by providing complete, geo-referenced resources under open licenses, making it highly valuable for future research.

Is the study design appropriate and does the work have academic merit?

Yes

Is the work clearly and accurately presented and does it cite the current literature?

Yes

If applicable, is the statistical analysis and its interpretation appropriate?

Yes

Are all the source data underlying the results available to ensure full reproducibility?

Yes

Are the conclusions drawn adequately supported by the results?

Yes

Are sufficient details of methods and analysis provided to allow replication by others?

Yes

Reviewer Expertise:

Geospatial sciences, open source software, research, food systems and data science

I confirm that I have read this submission and believe that I have an appropriate level of expertise to confirm that it is of an acceptable scientific standard.

# Reviewer report: Reviewer response for version 1

**DOI:** 10.21956/openreseurope.22012.r56676

**Published:** 2025-08-18

**Authors:** Vassilis Detsis

**Affiliations:** 1 Harokopio University, Athens, Greece

The paper presents a workflow to construct a georeferenced database from historical survey data in printed form. The objective was to make existing data available for computerised analyses. Such data predate the era of satellite images and even country-wide coverage of aerial photographs and are thus the only available country-wide window to view land cover and land use coverage in previous times. The survey data were scanned, read through an OCR application, organised in tables and linked to the province areas of the time of the survey. A number of maps and summary analyses showcase possible ways of utilisation of the data.

 The paper is well written and presented and, to the best of my knowledge, the cited literature is appropriate. The design is also appropriate and can be used in subsequent works using different kinds of data with or without spatial dimensions. A number of clarifications are due with respect to the methods and the terminology used that will be detailed below. The data, as well as the code used, are fully available. I have checked the datasets and maps, including plotting indicative attributes at random, and they look fine. I noticed a single point in the rainfall map being way off to the west, probably an error that should be corrected. I have not tried running the scripts since I am not familiar with Python. The Discussion and Conclusions are consistent with the Results.

 A single major concern is the fact that the CORINE land cover nomenclature is not used appropriately. Class 211 is Non-irrigated arable land, which might include ligneous crops (<25% of the area in question) but not applicable for agriforestry, which is class 244. However, the wording of the paper in the Results and the Discussion sections suggests that the talk is about fruit trees and not forestry trees, in which case class 211 is correct, although its description as agroforestry is not. If the latter is the case it should be corrected throughout the paper. Similarly, arable land belongs to classes 211 and 212 and not, as incorrectly stated, to 244. Class 311 does not refer to managed forest but to broad-leaved forest, managed or otherwise. Furthermore, absence of information is no argument to classify a forest as mixed (class 313). The author should leave it at the second level of the CORINE nomenclature, namely simply forest and class 31.

 The author could consider if it would be more productive to stick to the original categories of the survey instead of using the CORINE nomenclature. Linking the two categorisations involves a number of assumptions and it is necessary only if one intends to compare the historical land use and cover with the current one. If the author sticks to this reclassification a table should be provided clearly stating which of the categories listed in the “Extended_data” table is included in which CORINE category used to create the maps in figures 2 and 3. The assumptions underlying each link should also be presented.

 Some minor concerns related to methodological and related terminology issues are listed below, organised by Section:

 Methods

*This enabled the semi-automated extraction of tabular information, .....*: More information is due here. In which respect was the process not fully automated? The OCR applications are not flawless, furthermore the quality of their output depends on the condition of the printed materials. How was the output checked for errors?

*In parallel, modern provincial vector layers were sourced from the National Geodatabase and edited to reconstruct the 1929 territorial framework.*: This phrase is unclear. How were modern boundaries used to reconstruct the old ones and why, since the boundaries were already drawn from the survey itself, as described in the phrase immediately preceding this one.

*These predictions were output as a GeoTIFF file, providing a spatial representation of the 1920s precipitation distribution.*: The cell size should be mentioned.

 Results

 Define *quintal*, what is the equivalent in S.I. units?

 Figure 1. Units are missing. In table 1 of the extended_data the reader is informed that it is the number of animals, but it should be included in the caption or legend. The *total population* category, apparently created by the author from the raw data, could also be numbers or animal units. Please present the units.

 Figure 2. The caption would be more comprehensible if the table linking these categories to the original ones is provided (see the major concern above). Alternatively, the author could use the original survey categories.

 Figure 3. As above. Furthermore, while most categories are clear there is no indication of what *cash crops* might include.

 With respect to the argumentation I have no major concerns, two minor issues are listed below:

 In the Introduction it is stated that *In environmental modelling, LULC change data supports the prediction of future land use scenarios and the assessment of ecosystem impacts, ... *The statement is correct, but vague. These datasets are invaluable tools enabling an understanding of past changes. Since land systems exhibit path dependency they can indeed support prediction of future scenarios. On the other hand, the processes at work are different from the past and thus future scenarios cannot be simple projections of past changes into the future. So, while the argument is valid in principle, an elaboration on how data on past changes can support future predictions would be appreciated.

 In the Discussion it is stated that *In response to increased food demand, agricultural systems transitioned from extensive models, such as agroforestry, to more intensive methods of cultivation.* This is highly questionable. I am not familiar with the Italian context, however experience from elsewhere suggests that the driving force for intensification was the availability of capital to adopt the new technologies that became available in the international context already since the late 19 ^th^ century.

 All in all, this is a welcome contribution in the field of study of historical land systems. It provides a solid workflow along with necessary scripts and a complete geodatabase of the italian context in 1929. It thus enables the informed quantified study of land cover changes and comparisons with published past land cover maps modeled at the continental scale, enabling an assessment of their accuracy. The analyses undertaken highlight indicative potential uses, however a number of clarifications or modifications are due.

Is the study design appropriate and does the work have academic merit?

Yes

Is the work clearly and accurately presented and does it cite the current literature?

Yes

If applicable, is the statistical analysis and its interpretation appropriate?

Partly

Are all the source data underlying the results available to ensure full reproducibility?

Yes

Are the conclusions drawn adequately supported by the results?

Yes

Are sufficient details of methods and analysis provided to allow replication by others?

Partly

Reviewer Expertise:

Land systems science

I confirm that I have read this submission and believe that I have an appropriate level of expertise to confirm that it is of an acceptable scientific standard, however I have significant reservations, as outlined above.

# Reviewer report: Reviewer response for version 1

**DOI:** 10.21956/openreseurope.22012.r56667

**Published:** 2025-08-18

**Authors:** Giuseppe Cillis

**Affiliations:** 1 IMAA-CNR, Potenza, Italy

The article is well written and the methodology is quite clear. 

 The use and availability of this dataset is of fundamental importance for those involved in territorial transformations in Italy. 

 Obviously, it is an application exclusive to Italy, but the workflow can be applied in other territorial contexts where this type of data is available.

 To make it even easier to read, we suggest creating a graphical workflow diagram showing all the steps. In addition, the introduction should include a brief description of other Italian studies that have addressed this topic (studies covering the entire country, not just local or regional ones). It might also be useful to describe the type of agricultural cadastre in more detail. Information on its creation can be found in the ISTAT publication itself.

 In the conclusions, mention what other information can be obtained from the Cadastre and what the future implications are.

 In the conclusions, report what other information can be obtained from the register and what the future implications are.

Is the study design appropriate and does the work have academic merit?

Yes

Is the work clearly and accurately presented and does it cite the current literature?

Yes

If applicable, is the statistical analysis and its interpretation appropriate?

Yes

Are all the source data underlying the results available to ensure full reproducibility?

Yes

Are the conclusions drawn adequately supported by the results?

Yes

Are sufficient details of methods and analysis provided to allow replication by others?

Yes

Reviewer Expertise:

study of the historical dynamics of agricultural landscape transformation in a GIS environment

I confirm that I have read this submission and believe that I have an appropriate level of expertise to confirm that it is of an acceptable scientific standard, however I have significant reservations, as outlined above.
